# High genetic diversity of *Vibrio cholerae* in the European lake Neusiedler See is associated with intensive recombination in the reed habitat and the long-distance transfer of strains

**DOI:** 10.1111/1462-2920.13612

**Published:** 2017-01-18

**Authors:** Carina Pretzer, Irina S. Druzhinina, Carmen Amaro, Eva Benediktsdóttir, Ingela Hedenström, Dominique Hervio-Heath, Steliana Huhulescu, Franciska M. Schets, Andreas H. Farnleitner, Alexander K. T. Kirschner

**Affiliations:** 1 Medical University Vienna, Institute for Hygiene and Applied Immunology, Vienna, Austria; 2 Vienna University of Technology, Institute of Chemical Engineering, Vienna, Austria; 3 ERI BioTecMed University of Valencia, Valencia, Spain; 4 Faculty of Life and Environmental Sciences, University of Iceland, Reykjavík, Iceland; 5 Public Health Agency of Sweden, Solna, Sweden; 6 French Research Institute for Exploitation of the Sea (IFREMER), Plouzané, France; 7 Austrian Agency for Health and Food Safety (AGES), Vienna, Austria; 8 National Institute for Public Health and the Environment, Bilthoven, The Netherlands; 9 Interuniversity Cooperation Centre for Water & Health, (www.waterandhealth.at) Vienna, Austria

## Abstract

Coastal marine *Vibrio cholerae* populations usually exhibit high genetic diversity. To assess the genetic diversity of abundant *V. cholerae* non-O1/non-O139 populations in the Central European lake Neusiedler See, we performed a phylogenetic analysis based on *recA*, *toxR*, *gyrB* and *pyrH* loci sequenced for 472 strains. The strains were isolated from three ecologically different habitats in a lake that is a hot-spot of migrating birds and an important bathing water. We also analyzed 76 environmental and human *V. cholerae* non-O1/non-O139 isolates from Austria and other European countries and added sequences of seven genome-sequenced strains. Phylogenetic analysis showed that the lake supports a unique endemic diversity of *V. cholerae* that is particularly rich in the reed stand. Phylogenetic trees revealed that many *V. cholerae* isolates from European countries were genetically related to the strains present in the lake belonging to statistically supported monophyletic clades. We hypothesize that the observed phenomena can be explained by the high degree of genetic recombination that is particularly intensive in the reed stand, acting along with the long distance transfer of strains most probably via birds and/or humans. Thus, the Neusiedler See may serve as a bioreactor for the appearance of new strains with new (pathogenic) properties.

## Introduction

Environmental *Vibrio cholerae* populations represent a diverse genetic reservoir for the development of new epidemic clones (Rahman *et al*., 2008; Zhou *et al*., 2014). Until now, more than 200 serogroups have been described, with only two of them (O1 and O139) causing epidemic cholera. Moreover, some environmental *V. cholerae* strains possess a variety of virulence factors (e.g., haemolysin, zonula occludens toxin and type 3 secretion system) enabling them to induce severe infections other than cholera (Deshayes *et al*., 2015). The mechanisms of emergence and reasons for the observed high degree of genetic diversity are still under debate. Recent investigations indicated that genetic variability is linked to phenotypic polymorphism and that different phylogenetic lineages of *V. cholerae* in one environment occupy different ecological niches (Zo *et al*., 2008; Keymer *et al*., 2009). Shifts in crustaceaen zooplankton community structure, salinity and seasonal changes of abiotic factors like pH were the main drivers of micro-diversity of environmental *V. cholerae* populations in Chesapeake Bay, USA (Zo *et al*., 2008). Similarly, salinity, water temperature, and the presence of other organisms, as recently summarized in Takemura *et al*. (2014), have been reported to influence *V. cholerae* abundance and proliferation in the aquatic environment. Among these organisms, crustacean zooplankton (Huq *et al*., 1983; Heidelberg *et al*., 2002; Kirschner *et al*., 2008), planktonic algae (Islam *et al*., 1989; Eiler *et al*., 2007), prawns and oysters (Twedt *et al*., 1981) and also habitat structuring plants like macrophytes (Islam *et al*., 1989) and macroalgae (Vugia *et al*., 1997) serve as habitats for *V. cholerae* growth and biofilm formation. Thus, a high diversity of habitat structures and concomitantly the availability of different microecological niches can be hypothesized to promote genetic diversity of *V. cholerae*.

The Austrian steppe lake Neusiedler See, the largest endorheic lake in Central Europe covering 321 km^2^ offers ideal salinity (1–2 g L^−1^) and alkaline pH (8.5–9.1) conditions for *V. cholerae* proliferation (Kirschner *et al*., 2008). The lake is also a centre of the Austrian/Hungarian national parks Neusiedler See/Fertö-to, because it encompasses the largest bird protective area in Central Europe and it is an important bathing water. The open water area of the lake is surrounded by an extensive reed-stand almost equal in size providing a variety of habitat structures and a high degree of biodiversity (Schönberger, 2000), especially of birds (Nemeth *et al*., 2001). Halpern *et al*. (2008) hypothesized the important role that waterfowl (Anatidae) may play in *V. cholerae* transport from endemic areas to new habitats. This possibility was demonstrated by studies on faeces of healthy birds where O1 as well as non-O1 *V. cholerae* have been found (Lee *et al*., 1982; Ogg *et al*., 1989). *V. cholerae* living on crustaceans, molluscs and plants may be ingested by birds and/or transported through mud attached to the bodies of long distance migratory cormorants (Phalacrocoracidae) (Halpern *et al*., 2008), geese (Anserini), teals (*Anas* spp.) or sandpipers (Scolopacidae). During the relatively long warm part of the year, humans also intensively use the lake Neusiedler See for recreational activities. Several infections with *V. cholerae* non-O1/non-O139, mostly cases of otitis and one lethal sepsis, were registered in patients where a visit at lake Neusiedler See was documented (Huhulescu *et al*., 2007).

The aim of our study was to assess the genetic diversity of *V. cholerae* strains in three distinct habitats of the lake Neusiedler See – the open water area, the reed stand and an intermediate habitat – and link the observed patterns to key environmental parameters. A total set of 472 environmental strains was collected over a period of two years isolated from both water and zooplankton samples. Multilocus sequence analysis was performed based on the nucleotide alignment of four unlinked loci of the chromosome I – *gyrB*, *pyrH*, *recA* and *toxR*. In addition, eight clinical isolates from patients for whom a prior visit at the lake Neusiedler See was documented as well as the respective sequences of seven fully sequenced O1 strains from reference databases. To look for potential Pan-European transfer events of *V. cholerae* strains to the lake, 68 *V. cholerae* non-O1/non-O139 isolates (environmental and human) from six other European countries were included in the analysis.

## Results

### Ecological conditions at the reed stand habitat are significantly different compared to the open water and the intermediate habitat

There were significant differences in the physicochemical characteristics between the three investigated habitats of the lake Neusiedler See (Table 1). Total suspended solids and total phosphorus values were significantly lower in the reed stand in comparison to the open water and the intermediate habitat (analysis of variance, ANOVA; Tukey’s post hoc test; *P* < 0.001). Also pH values were significantly lower there (ANOVA; *P* < 0.001), but with a mean difference of 0.3 units, the observed difference can be considered negligible. Significantly higher concentrations of dissolved organic carbon were recorded in the reed stand, with a higher ratio of high molecular weight substances, reflected by a significantly lower 250/365 nm absorbance ratio (ANOVA; *P* < 0.001). In the reed habitat, the average numbers of copepods were higher than the number of cladocerans, while in the other habitats, it was vice versa. In addition, maximum zooplankton abundances were observed in the reed stand in winter and spring, while at the two other habitats zooplankton peaked in spring and summer (data not shown). Chlorophyll a values were lower in comparison to the open lake while total bacterial cell numbers were higher (ANOVA; *P* < 0.05). Thus, ecological characteristics indicated significant differences of the reed stand in comparison to the other two habitats. No differences between the habitats were observed for conductivity and water temperature (ANOVA; *P* > 0.1). Result of a cluster analysis based on the values for the discriminating variables is given in Fig. S1: the ‘intermediate habitat’ indeed represents the transition in terms of ecological properties between the open water and the reed habitat.

### Planktonic culturable V. cholerae showed equally high abundances at all three habitats

Culturable *V. cholerae* abundances ranged from not detectable in winter to ~100 × 10^3^ CFU L^−1^ in July 2012 at the intermediate habitat, where approx. 45% of all *V. cholerae* were attached to zooplankton (Schauer *et al*., 2015). With this exception, the majority of all other *V. cholerae* was in the planktonic state and not attached to zooplankton. High numbers (above 50 × 10^3^ CFU L^−1^) were observed at all three habitats without significant differences (ANOVA; *P* > 0.1). Details on the concentrations and distribution of *V. cholerae* on zooplankton and in the water phase in this lake were published elsewhere (Schauer *et al*., 2015). All isolates that were picked and tested positive for *V. cholerae* with PCR were negative for the presence of *ctx* and *tcp* genes.

### Polymorphism of the phylogenetic markers used for multilocus sequence analysis

Nucleotide properties of selected phylogenetic markers and their suitability for phylogenetic analysis were first tested for fragments of the four housekeeping genes located on chromosome I encoding DNA gyrase subunit B (*gyrB*), recombinase A (*recA*), uridylate kinase (*pyrH*), DNA-directed RNA polymerase subunit alpha (*rpoA*) and the virulence associated gene cholera toxin transcriptional activator (*toxR*), respectively, that were obtained for the first 299 *V. cholerae* isolates recovered from the lake on a single day in July 2012. From the five selected genes, *recA* and *toxR* showed most of the segregating sites, while *pyrH* and *gyrB* were less phylogenetically informative (Table 2). The *rpoA* gene failed to differentiate sufficiently between the isolates showing <10 of segregating sites for the entire dataset (data not shown). Therefore, it was excluded from the investigation, and the remaining *gyrB*, *recA*, *pyrH* and *toxR* were used for multilocus sequence analysis (MLSA). Tajima’s *D* test showed neutral evolution for all loci but *recA*. In the latter gene, a significant negative value was obtained indicating the presence of possible purifying selection and/or population size expansion (Tajima, 1989). To distinguish between these two phenomena, we tested the population size parameters based on all other loci (Table 2). We detected an excess of low frequency polymorphisms being present in the three other loci – *gyrB*, *toxR* and *pyrH*, which resulted in negative (albeit insignificant) Tajima’s *D* values for them as well. Consequently we assume that the significant Tajima’s *D* value for *recA* is rather explained by growing population size than by natural selection pressure in this locus. We therefore considered the use of locus *recA* for MLSA in the concatenated dataset together with the three other loci as permissible (Table S1). The applicability of *recA* for MLSA was also demonstrated previously (Garg *et al*., 2003; Kotetishvili *et al*., 2003; Thompson *et al*., 2004; Mohapatra *et al*., 2009; Teh *et al*., 2011; Thompson *et al*., 2011). The resulting alignment, thus, included four phylogenetic loci with 2270 bp in total.

### Reed stand habitat supports highest genetic diversity of V. cholerae

**Fig. 1:**
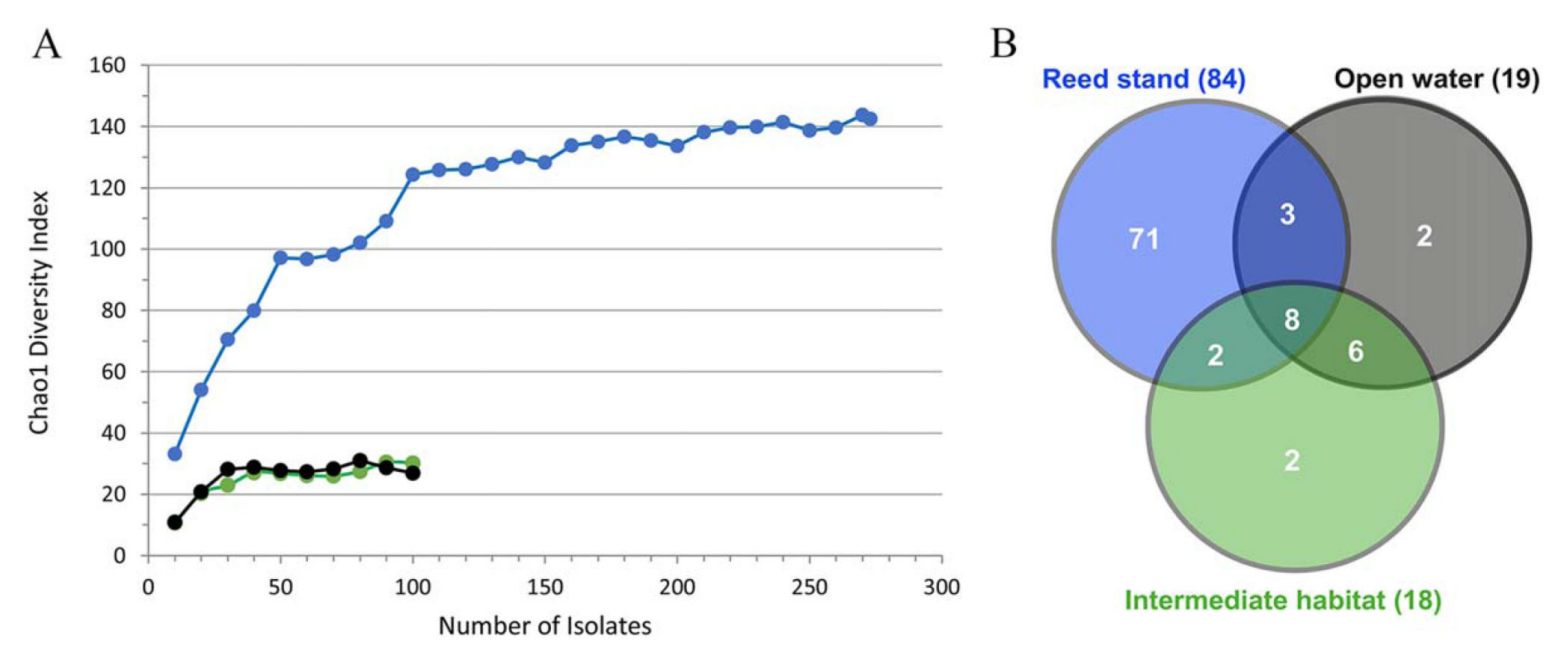
A. Chao1 curves to depict the genetic diversity of *V. cholerae* and the depth of sampling at three habitats based on the used OTU definition (sequence type). Blue line: reed-stand, black line: open water and green line: intermediate habitat. For the open water and intermediate habitat, 100 *V. cholerae* strains were isolated on a single day in July 2012, for the reed stand habitat, additional 173 strains from seasonal sampling (2011 + 2012) were analyzed. B. Venn diagram of the number of sequence types based on the entire dataset of samples of the lake Neusiedler See. The three circles indicate the habitats and the numbers correspond to the quantity of unique or shared sequence types. Total numbers of sequence types for each habitat are noted in brackets.

To estimate the genetic diversity of *V. cholerae* isolates in the three habitats we initially analyzed the first dataset that included the alignment of the four loci for 299 (ca. 100 per habitat) strains isolated from the lake on a single day in July 2012. Thirty-two sequence types were found among 100 strains isolated from the reed stand habitat. In contrast, only 18 and 19 sequence types occurred in the intermediate and the open water habitats, respectively. Among the 32 sequence types of the reed stand, 20 were unique to this habitat, while in the open water and the intermediate habitat, only two unique sequence types were identified (data not shown). The rarefaction curves based on Chao1 diversity richness index indicated a saturation of the diversity for all three habitats with the respective sample size (Fig. 1A) based on the used OTU definition (sequence type). However, due to the high degree of diversity at the reed stand saturation was obtained only after including additional 173 additional strains from this habitat isolated from water (*n* = 116) and zooplankton (*n* = 57) between April 2011 and November 2012. The inclusion of these data indicated that at least 140 sequence types are present in this habitat over two annual cycles, from which 84 sequence types were found (Fig. 1B). In total, for the increased data set of the lake, 94 sequence types were observed. A total of 71 were unique to the reed stand, two sequence types were unique to the intermediate habitat and to the open water habitat, and 19 were shared between the different habitats (Fig. 1B).

The diversity of *V. cholerae* was estimated using the frequency of every sequence type in individual habitats. The Shannon biodiversity index showed essentially higher values for the reed stand habitat (initial and enlarged datasets), while open water and the intermediate habitat were similar (Table 3). The high diversity in the reed-stand was confirmed by the Berger–Parker Dominance index that showed the high importance of the major sequence types in open water and in the intermediate habitat while it was considerably lower in the reed stand (Table 3). Thus, all indexes of alpha-diversity demonstrate that the reed habitat has the highest genetic diversity of *V. cholerae* strains in comparison to the two other habitats.

### Multilocus phylogenetic analysis of V. cholerae isolates from the lake Neusiedler See

**Fig. 2:**
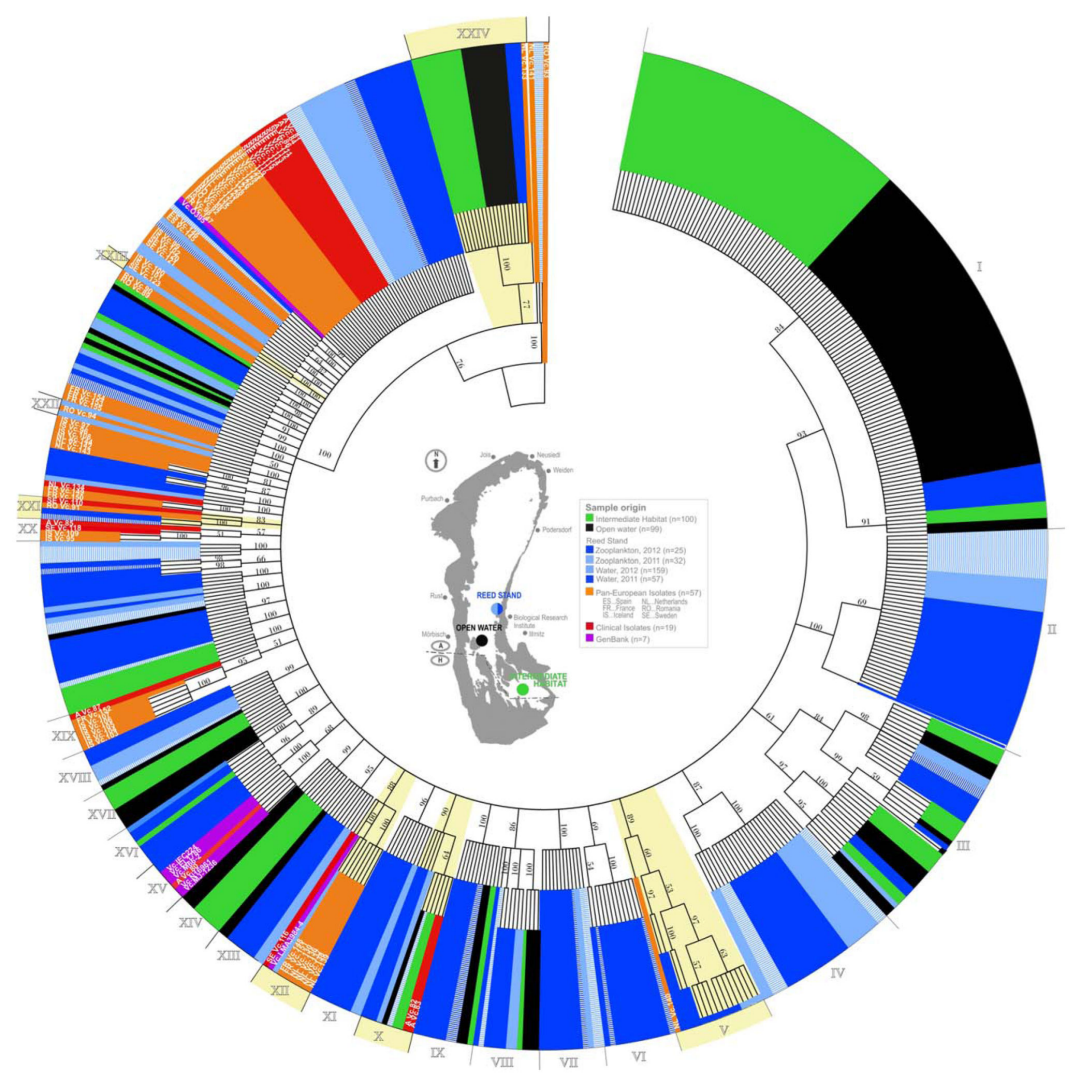
Maximum likelihood phylogenetic tree resulting from the analysis of the concatenated sequences comprising of gyrB, recA, pyrH and toxR (2270 bp) of 555 *Vibrio cholerae* isolates including 472 of lake Neusiedler See, 68 Pan-European isolates, 7 reference strains and 8 clinical isolates. Numerical values at nodes indicate bootstrap support (>50) obtained after 1000 replications. Clades discussed in text are given in Roman numerals. Colors denote origin of isolate detailed on the insert. Clades with mixed local and remote isolates (V, XII, XXI, XXIII and XXIV) and with mixed local environmental and clinical isolates (X) are marked with yellow. Insert depicts a schematic map of the lake, location of studied habitats and the sample type (water, zooplankton). Branch lengths are transformed to equal length for better visualization and do not represent true phylogenetic distances.

The phylogenetic analysis of the concatenated sequences of the four loci for 480 Austrian (472 environmental and eight clinical) and 75 international isolates (555 in total) revealed a complex and highly resolved phylogenetic tree (Figs. 2 and S2). For the Maximum Likelihood tree, it revealed 50 statistically supported clades (bootstrap value >70), with at least two isolates per clade that included about 90% of the isolates. Among them, 36 clades were formed exclusively by isolates from the lake Neusiedler See, five clades were mixed and contained isolates from the lake and from the remote habitats and nine clades were composed of exclusively Pan-European isolates. In Fig. 2, only clades (24) that either consist of more than six strains or are composed of mixed isolates from different countries were given Roman numerals (I–XXIV).

Most apparently, several of the large clades from the lake that did not contain international strains always occupied terminal positions on the phylogenetic tree (e.g., clades I, II, III, IV, VI, VIII and XVI), suggesting unique endemic diversity of *V. cholerae* in this ecosystem. In line with the results of biodiversity assessments (see above), all lake isolates from open water and from the intermediate habitat belonging to numbered clades also contained strains from the other habitats. In contrast to these ‘mixed’ local clades, there was a number of clades formed exclusively by strains from the reed stand (II, IV, VI, VII, XI and XVIII) supporting the outstandingly high diversity of *V. cholerae* in this habitat. In the reed stand clades, isolates from zooplankton and water were usually mixed together, suggesting no genetic difference between *V. cholerae* inhabiting one or another ecological niche. Similarly, no genetic difference between isolates from 2012 and 2011 could be detected.

Five supported clades contained international isolates mixed with local isolates from the lake (clades V, XII, XXI, XXIII and XXIV). The largest of these clades is clade V, where 16 strains from the reed-stand cluster with a strain from the Netherlands. In the other mixed clades, strains from Sweden (clades XII, XXI and XXIII), the Netherlands (clades XII and XXIV), Romania (clade XXI) and France (clade XII) were mixed with the Austrian strains. The Swedish strain VC123, for example, differed from the Austrian strain in the common clade XXI by only one nucleotide. No common clades with the strains from Spain and Iceland were observed. A special case was observed for clade XV, where clinical O1 strains (including one from Austria) cluster with two Austrian strains from the reed-stand. However, for this clade, the bootstrap value of 68 was slightly below 70 and, thus, not statistically supported. In another clade X, two Austrian clinical strains (A VC82 and A VC83) isolated from patients in 2008 and 2009, respectively, had identical sequences like strains isolated from the lake in 2011 and 2012.

Among the 555 studied isolates, 25 strains (22 from the lake, two from the Netherlands and one from Romania, including clade XXIV and three additional strains; Fig. 2) were shown to be distant and occupied the basal position to the large monophyletic clade that comprised most of the diversity of *V. cholerae*.

All the findings based on the maximum likelihood method described above were also supported by the Bayesian approach (*P* > 0.94). The Bayesian tree can be found in Fig. S2.

### Indications for the long distance transfer of *V. cholerae* isolates in Europe

The phylogenetic analysis of the concatenated dataset revealed that many international isolates showed genetic relation to the strains from the lake (i.e., belonged to common monophyletic clades; see above), almost no terminal clades that contained both Austrian and Pan-European strains were detected (Figs. 2 and S2). Such topology of the concatenated tree can be explained by the cumulative action of the long distance transfer of *V. cholerae* (=introduction of ancestral alleles) and the intensive genetic recombination taking place in the lake (=formation of unique sequence types). To corroborate this hypothesis, we examined topologies of the individual loci trees (Figs. S3 and S4). Contrary to the structure of the concatenated phylogenetic tree, the most polymorphic loci *recA* and *toxR* trees revealed an almost homogenous mixture of Pan-European isolates with strains from the lake Neusiedler See. Among 21 statistically supported terminal clades for *recA*, seven contained isolates from the lake together with international strains. Seven such mixed clades were also detected on the *toxR* phylogenetic tree. Although the resolution of the *gyrB* and *pyrH* phylogenetic trees is generally low in comparison to *recA* and *toxR*, mixed terminal clades composed of local and Pan-European isolates were also present (Fig. S4). These findings show that isolates from the lake share numerous individual alleles with strains isolated from such remote locations as Sweden, the Netherlands, France and Romania and a panmictic population structure, that is, members of the population move freely across habitats, likely are able to exchange DNA and share a common gene pool (Hoffmann *et al*., 2010). Such phenomenon may be linked to the action of the long distance transfer between habitats by birds or humans.

### Reed stand habitat supports intensive genetic recombination between V. cholerae isolates

**Fig. 3:**
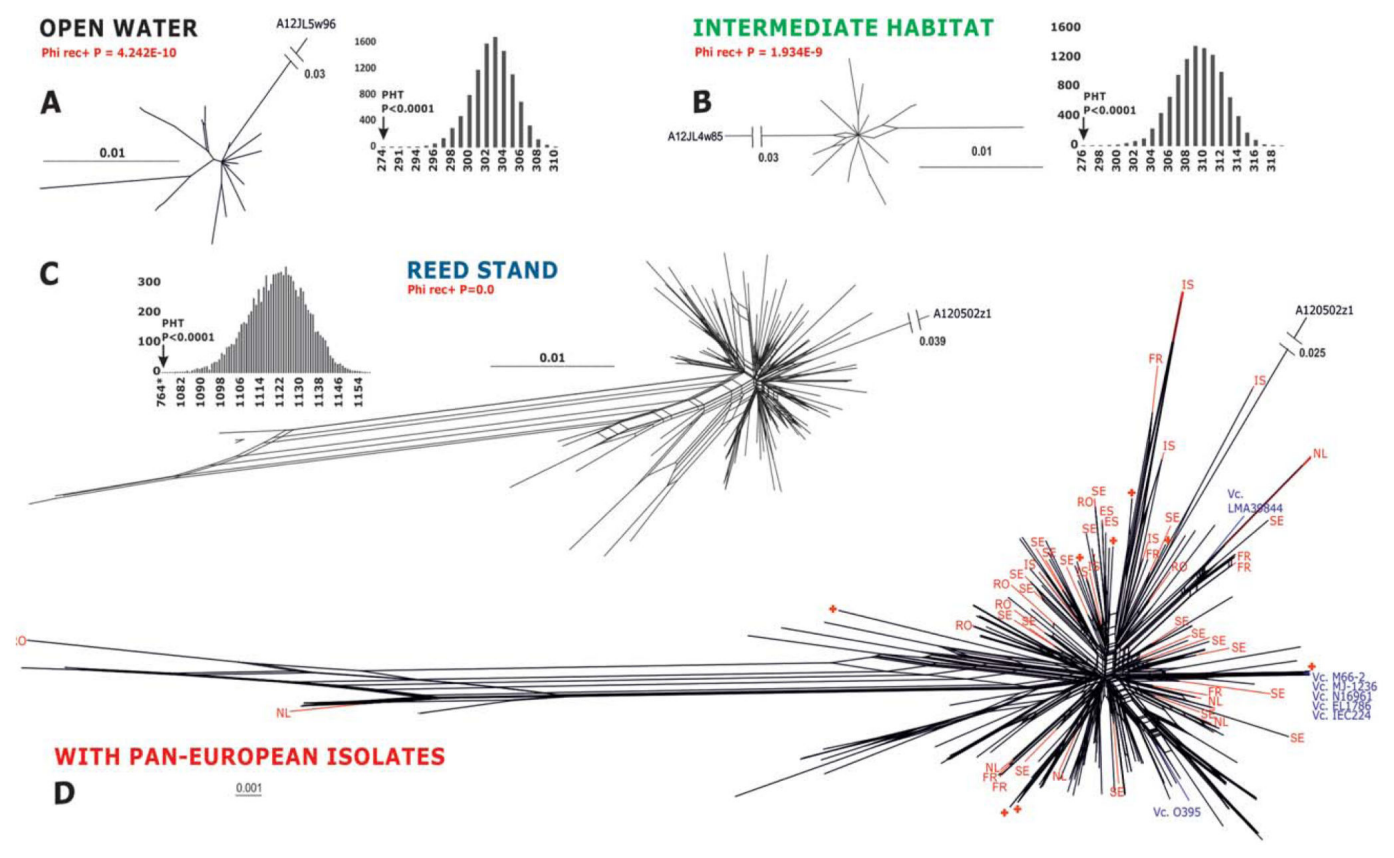
Reconstruction of possible recombination networks between *V. cholerae* isolates from each habitat (A–C) from the lake Neusiedler See and between the total population of the lake and reference Pan-European strains (D). The split decomposition method was applied to the concatenated dataset of four genes. Gaps were treated as missing characters throughout. Bar plots on (A)–(C) indicate results of partition homogeneity tests (PHT) for *V. cholerae* isolates from each habitat. Arrows indicate the actual summed tree length and corresponding *P*-value. For PHT, parsimony-uninformative characters were excluded, gaps were treated as missing characters, and 10 000 repetitions of heuristic search under parsimony criterion were performed. Results of phi-test and respective *P*-value as implemented in SplitsTree based on the pairwise homoplasy index, PHI (=*F*) statistic, to detect refined incompatibility indicating recombination is shown for (A)–(C). rec+ indicates statistically significant support for recombination.

In order to test whether *V. cholerae* isolates from the distinct phylogenetic clades revealed for each of the three habitats are separated by a recombination barrier, we used the split decomposition method provided by the SplitsTree package (Huson and Bryant, 2006). Using a concatenated dataset of the four loci, this analysis enabled us to test for the presence of network relationships within isolates from each habitat and also test the recombination/isolation between local and remote strains. This method presents conflicting phylogenetic data, presumably arising from recombination, as an interconnected network of lineages. Such a network was evident for all three individual data sets (Fig. 3A–C) and for the total data set including Pan-European isolates (Fig. 3D). The comparison of these networks documented an outstanding complexity for the reed stand compared to relatively simple networks in open water and the intermediate habitat. Interestingly, this network contained no separated clonal groups that could correspond to individual phylogenetic clades seen in Fig. 2 indicating the high level of genetic recombination. When the PHI test implemented in the same software package (Huson and Bryant, 2006) was applied to the data sets from the individual habitats, statistical significance for the presence of the recombination was obtained for each case (Fig. 3) having the lowest *P*-value for the reed stand. These data indicate that the latter habitat is not only the most diverse but also supports the highest intensity of genetic recombination. To this end, the analysis of all Austrian isolates together with Pan-European strains is particularly interesting as it reveals no geographic groupings but a homogenous network with isolates from the lake clustering together with other European (and frequently remote) strains. This analysis corroborates the hypothesis of the effective long-distance transfer of *V. cholerae* isolates at least on the European continent.

To verify this conclusion by another method, the partition homogeneity test (Huelsenbeck *et al*., 1996) was used to examine the congruence between gene trees. In this test, artificial data sets are produced by multiple (10.000) resampling and random swapping of observed data sets and subsequent construction of maximum parsimony trees for every newly sampled ‘gene’ sequence. For nonrecombining populations, the sums of the lengths of the gene trees for the observed and resampled data should be similar, but under recombination the sums of the tree lengths should be longer than that for the actual data because recombination among unlinked sites should introduce homoplasy into the data. This analysis revealed topological conflicts present in all three habitats (Fig. 3A–C), thus providing another evidence for the presence of genetic recombination between respective *V. cholerae* strains. In order to verify this result by alternative means, the maximum Chi-square test of Smith (1992), linkage disequilibrium (LD) *r*^2^ (Hill and Robertson, 1968) and LDD0 (LD versus distance |*D*|; Lewontin, 1964) estimates available on RecombiTEST webpage (see ‘Experimental procedure’ section) also detected recombination between strains of all individual habitats (data not shown).

Thus, alternative methodological approaches resulted in detection of genetic recombination that takes place in all three habitats of the lake Neusiedler See. Considering the comparable abundance of *V. cholerae* in these habitats the values obtained for the reed stand indicate that it provides conditions favouring outstandingly high diversity of *V. cholerae* accompanied by intensive genetic exchange between the strains.

## Discussion

### A complex habitat structure in the reed stand boosts V. cholerae diversity through high level of genetic recombination

The affinity of environmental populations of *V. cholerae* and related taxa to genetic recombination within and between species has been documented in several studies (Salim *et al*., 2005; Feng *et al*., 2008; Keymer and Boehm, 2011; Octavia *et al*., 2013; Esteves *et al*., 2015; Orata *et al*., 2015). Orata *et al*. (2015) have demonstrated cases of genetic interactions between *V. cholerae* and its sympatric sister species *V. metoecus*. Their study documented that 24% of core genes including those involved in pathogenicity show signals for interspecific recombination. Thus, the virulence factors of *V. cholerae* are more widespread than previously believed. Esteves *et al*. (2015) concluded that the recombination may have a major impact on the diversification of lineages and thus on the emergence of novel pathogenic strains. Their results demonstrated frequent recombination among such pathogenic species as *Vibrio parahaemolyticus*, *V. cholerae*, *V. mimicus* and the abovementioned *V. metoecus*. Interestingly, the correlation between environmental conditions and recombination frequency documented that in lower salinity conditions the recombination was less apparent (Esteves *et al*., 2015).

In this study, we collected a large sample size and investigated an infraspecific population of *V. cholerae* in the moderately saline lake Neusiedler See. Our results demonstrate that despite the equal abundance of *V. cholerae* isolates in different habitats across the lake, the diversity of *V. cholerae* is significantly higher in the reed stand compared to open water. The reasons for the outstandingly high diversity in reed stand habitat may be (i) ecological, implying that this ecosystem offers a diversity of microecological niches leading to diversifying natural selection and to higher rates of genetic recombination between *V. cholerae* isolates, and (ii) biological, assuming that this habitat represents a natural hub for diverse sequence types of *V. cholerae* that emerged in remote regions and were transferred here, or/and interaction of these factors. The reed stand habitat (a small pond within the reed stand) is highly structured. It is sheltered from wind induced sediment resuspension allowing growth of submerged vegetation and algae. The water column is not efficiently homogenized by the action of wind and the pond is surrounded by emergent reed vegetation of different age, the submerged parts of which act as substratum for the growth of epiphytic and saprotrophic organisms. It has been reported that *V. cholerae* is able to form biofilms on a wide range of biotic surfaces and it has been isolated from algae and aquatic vegetation (Islam *et al*., 1989). The reed stand of the lake is also part of an important bird sanctuary (national park Neusiedler See – Seewinkel/Fertö-to) with approximately 350 described bird species (Dvorak *et al*., 2014) and with >50 species occurring in the reed stand. In addition, a large variety of other animals (fish, amphibians, molluscs and other invertebrates) are present. Especially birds have been hypothesized to act as possible vectors of large-distance transfer of *V. cholerae* (Halpern *et al*., 2008) that could add to the observed high genetic diversity of this bacterial species in the reed stand. The open lake water on the other hand, as well as the water at the intermediate habitat, experience permanent homogenization due to the action of wind, as the lake is very shallow (max. depth 1.8 m). Due to the concomitant resuspension of fine sediment, the water column is highly turbid preventing the growth of submerged vegetation and algal blooms. This leads us to conclude that it is the specific habitat structure that represents the crucial factor for the observed differences in *V. cholerae* diversity between habitats.

Cases of high genetic diversity were already reported for environmental *V. cholerae* populations in coastal waters and sediments from the central California coast (Keymer and Boehm, 2011) and in Mediterranean lagoons (Esteves *et al*., 2015). In those studies, a limited number of isolates were analyzed for each sampling site at each time point. Thus, the whole population structure present at a specific site on a specific time point was not depicted. Our study showed that in order to get a representative picture of the *V. cholerae* population structure in an aquatic environment suitable for these bacteria, several dozens of isolates have to be analyzed per sampling event. From the rarefaction curves, it became clear that even in the rather homogenous open water habitat at least 30–40 isolates have to be analyzed to reach saturation at the level of sequence types (the OTU definition that was used in this study). In the reed-stand habitat with the highest *V. cholerae* diversity, a sampling depth of approx. 250 isolates per site would be necessary. When comparing the three investigated habitats of the lake Neusiedler See, it became evident that the reed-stand habitat displayed by far the highest *V. cholerae* genetic diversity, corroborated by all calculated diversity indices. On a single day in July 2012, thirty-two sequence types were found among the 100 isolates, with 20 being unique to this habitat. Within the increased data-set, including seasonal strains from water and zooplankton isolated from the reed stand habitat, 84 sequence types of in total 273 analysed strains were found. In contrast to this, the number of sequence types in the open water and intermediate habitat was much lower (19 and 18 out of 99 and 100 isolates, respectively) and only two unique sequence types were observed for those habitats. In a recent study, Kirchberger *et al*. (2016) were analyzing 438 isolates via MLST from a coastal pond and lagoon system sampled at two single dates, and interpreted that the observed population was highly clonal. Among the 438 isolates, 85 different sequence types were found with 95% of all strains belonging to 17 clonal complexes (i.e., clades). Compared to this, for the 472 strains from the lake Neusiedler See the number of clades was 41 (without the pure ‘international’ clades) with a slightly higher number of 95 sequence types derived mostly from sampling the reed habitat over two consecutive years. Thus, the higher number of clonal complexes (clades) in our study in comparison to the study of Kirchberger *et al*. (2016) indicates a higher diversity of the *V. cholerae* strains in the lake. However, without sampling the complex reed habitat, the diversity of the lake's *V. cholerae* population would have been erroneously underestimated.

Interestingly, no significant differences between the *V. cholerae* populations isolated from zooplankton and from water in the reed stand habitat were found. In addition, no significant differences between the *V. cholerae* populations isolated in 2011 and the ones isolated in 2012 were observed. In all cases, isolates were evenly spread over the phylogenetic trees. Due to the fact that only 57 zooplankton isolates from the reed-stand were analysed (in comparison to 216 isolates from the water), it cannot be excluded that potential differences were masked by ‘under-sampling’ the *V. cholerae* diversity on zooplankton. In our opinion, a reliable assignment of *V. cholerae* population dynamics to the effects of specific environmental factors is only valid, if the number of strains isolated per sampling event is representative of the total population, a factor that has often been neglected in other studies (e.g., Schuster *et al*., 2011).

Although the presence of genetic recombination between *V. cholerae* and sister species has been documented (see above), in most of the studies, sequence types of several related species were analyzed. Keymer and Boehm (2011) investigated 161 isolates of *V. cholerae* and eight strains of the three related species (*V. parahaemolyticus, Vibrio alginolyticus* and *Vibrio aestuarianus*). Although their recombination tests gave strongly positive signals, the topologies of the trees were considered congruent and concordant to the difference between species. In our study we exclusively analysed isolates of *V. cholerae* and revealed both intensive recombination present in all habitats but more effective in the reed stand and also resulting incongruence between the topologies of the single loci trees.

### The V. cholerae populations in the lake are linked to clinical and Pan-European isolates

It was surprising to see that two Austrian clinical isolates from 2008 and 2009 had the same sequence type as four strains isolated from all three lake habitats in 2012. These results most likely confirm that the patients got their *V. cholerae* infections from recreational activities in the lake and show that specific (pathogenic) strains or their descendants are present in the lake over several years at multiple sites.

Even more surprising, there were many large monophyletic clades that combined Austrian environmental isolates with environmental and clinical isolates from several European countries except Iceland and Spain. The analysis of the individual gene trees revealed that isolates from the lake shared numerous individual alleles with strains isolated from such relatively remote regions like Sweden, Netherlands, France and Romania, indicating a panmictic population structure of the Vibrios (Hoffmann *et al*., 2010). In an attempt to explain this observation we propose the long-distance transfer of strains via birds or humans. It has been already mentioned above that the lake Neusiedler See is part of the largest bird protective area in Central Europe and that a high number of different migrating bird species are encountered in this region. Migrating birds were hypothesized to play a potential key role in the long distance transfer of *V. cholerae* across continents (Halpern *et al*., 2008) and waterfowl was shown to carry O1 as well as non-O1 *V. cholerae* in their faeces (Lee *et al*., 1982; Ogg *et al*., 1989).

Five of the seven strains (*V. cholerae* O1 isolated from India, Bangladesh and Brazil), for which reference sequences were obtained from NCBI GenBank, had the same sequence type as an Austrian clinical isolate (a laboratory accident) that was also a *V. cholerae* O1 strain. These strains formed a common clade (clade XV) with two strains isolated from the lake Neusiedler See that was supported in the Bayesian tree and only slightly below the level of significance in the maximum likelihood analysis. In another clade (clade XII), an environmental nontoxigenic O1 strain LMA3984-4 isolated in Brazil (Perez Chaparro *et al*., 2011; Sa *et al*., 2012) clustered with a Swedish clinical strain, environmental strains from France and the Netherlands and an isolate from lake Neusiedler See. These findings strongly indicate a panmictic population structure of *V. cholerae* non-O1/non-O139, probably caused by the long-distance transport of strains that has also occurred across continental borders.

## Conclusion

Our results show that endorheic saline steppe lakes, that are similar to Neusiedler See in Austria, may harbour highly diverse populations of *V. cholerae*, linked mainly to specific highly structured habitats like a reed stand. The diversity is well explained by intensive genetic recombination within local strains and between them and strains imported through the long distance transfer (most probably via birds or humans). Considering that such lakes are usually important bathing waters, we highlight their significance for public health because these ecosystems may serve as bioreactors for the appearance of *V. cholerae* strains with novel properties including pathogenicity.

## Experimental procedures

### Study site, sampling and strain collection

#### Study site

The lake Neusiedler See is the largest shallow alkaline lake in Central Europe (surface area 321 km^2^; maximum depth 1.8 m; mean depth 1.1 m; pH 8.5–9.1, salinity: 1–2 g L^−1^). A reed-stand composed of *Phragmites australis* covers 55% of the total lake area. The lake is an important bathing water.

#### Sampling

Water samples were collected in July 2012 from three different habitats: from open water, from a small area of water entirely enclosed by reed stand and from a window of open water in vicinity of the reed stand (named ‘intermediate habitat’, based on chemophysical and structural properties). Samples were taken by boat in triplicates with clean, sterile 500 mL glass-bottles at a water depth of 30 cm. For the reed stand habitat, additional water and zooplankton samples were included in the analysis, taken in biweekly to monthly intervals from April 2011 to November 2012. As described in detail elsewhere (Kirschner *et al*., 2008), crustacean zooplankton samples were collected with vertical net hauls (mesh size, 250 μm) resulting in integrated samples of the entire water column. Depending on water depth at the respective station (75–120 cm), 50–80 L of lake water was filtered. Zooplankton was collected from the net and transferred into sterile 200 mL glass bottles. All samples were transported to the laboratory in a cooling box at the measured *in situ* temperature (±3°C).

#### Environmental variables

A variety of abiotic environmental variables (water temperature, pH, electrical conductivity, total phosphorus, chlorophyll a, dissolved organic carbon, total suspended solids, 250/365 nm absorbance ratio as an indicator for total aromaticity and the presence of humic substances) were determined according to Kirschner *et al*. (2008). Total bacterial cell numbers were determined by SybrGold staining according to a modified protocol of Riepl *et al*. (2011).

#### Isolation of *V. cholera*

Different volumes from 1 mL up to 100 mL of the water samples were filtered through 0.45 μm pore-size cellulose nitrate filters (Ø 50 mm; Sartorius Stedim Biotech, Göttingen, Germany), subsequently placed on Thiosulfate Citrate Bile Sucrose agar plates (TCBS; Merck, Darmstadt, Germany) and incubated for 18 h at 37°C. Yellow, round, 1–3 mm diameter colonies were counted, and 10 representative colonies were picked and streaked onto nutrient agar plates without NaCl (3% beef extract, 5% peptone and 15% agar) and incubated for 24 h at 37°C. Grown cultures were considered presumptive *V. cholerae* (Schauer *et al*., 2012) and were confirmed by species-specific *ompW*- PCR as described in Nandi *et al*. (2000). In addition, all isolates were tested for the presence of *ctx* and *tcp* genes using multiplex PCR (Goel *et al*., 2007).

A known amount of crustacean zooplankton individuals (mainly *Arctodiaptomus spinosus* and *Diaphanosoma mongolianum* (Kirschner *et al*., 2008)) were ground with a sterile glass micromortar and pistil, and suspended in 500 μL 1× PBS. After treatment for 20 min in an ultrasonic water-bath (Bandelin Sonorex Berlin, Germany), 150 μL of a 10-fold dilution series in 1× PBS were plated on TCBS agar plates and processed as described above.

#### Clinical and Pan-European samples

Eight *V. cholerae* samples isolated from patients in the years from 2008 to 2012 were provided by the Institute of Medical Microbiology and Hygiene, Austrian Agency for Health and Food Safety (AGES). Six of the isolates have been identified as *V. cholerae* non-O1/non-O139 related to patients visiting the lake Neusiedler See, and two (*Vc*. 80 and *Vc*. 87) were an O1 subtype Ogawa strain from a laboratory accident (Huhulescu *et al*., 2010), and an O1 subtype Inaba from a stay in Egypt. In addition, *V. cholerae* non-O1/non-O139 strains from patients and environmental samples were isolated by partners in six European countries. One human isolate and 14 environmental *V. cholerae* strains from the Wadden Sea and Binnenschelde were isolated between 2009 and 2012 in the Netherlands using a protocol described in detail in Schets *et al*. (2011). Fifteen strains were isolated from water, sediment, brown algae and invertebrates collected at three different regions in Iceland according to Haley *et al*. (2012). Two *V. cholerae* strains were isolated on TCBS agar plates after enrichment in alkaline peptone water from Alfacada pond in the Ebro delta (Tarragona, Spain) in June 2013. The species was identified by API20E. Ten environmental strains were isolated in 2006 from the Baltic Sea and nearby ponds in Southern Sweden. Samples were enriched in alkaline peptone water and streaked on TCBS agar plates. Species were identified by API 20E. In addition, 10 strains were isolated from patients experiencing wound, ear and bloodstream infections during the same period. Nine environmental strains were isolated from mussels and clams at the French Atlantic coast in 1999 and 2012 according to the protocol described in Hervio-Heath *et al*. (2002). Five strains were isolated from surface waters and wastewater in Romania between 1997 and 2002, after enrichment in alkaline peptone water and streaking on TCBS agar plates. The species was identified by API 20E. Additionally, two strains were isolated from human stools by the same procedure.

#### Reference strains

The genome sequences from seven different *V. cholerae* strains, available at the National Centre for Biotechnology Information (http://www.ncbi.nlm.nih.gov), were included in the phylogenetic analysis.

An overview and a detailed list of all strains used in this study can be found in Tables S2 and S3.

### MLSA

In MLSA, the fragments of housekeeping genes are usually used as phylogenetic markers to identify and subtype species and to perform evolutionary reconstructions (Thompson *et al*., 2005; Keymer and Boehm, 2011; Schuster *et al*., 2011). Virulence genes, or virulence-associated genes, are directly linked to the survival of the populations and therefore diversify more rapidly and are usually more polymorphic (Teh *et al*., 2011). They are therefore informative to discriminate better between specific clones (Cooper and Feil, 2004) and have been used in multiple studies (Garg *et al*., 2003; Kotetishvili *et al*., 2003; Lee *et al*., 2006; Teh *et al*., 2011). For obtaining an appropriate resolution of the genetic diversity of *V. cholerae*, we selected the four housekeeping genes, *recA*, *gyrB*, *pyrH* and *rpoA*, that are often used for phylogenetic analysis of *V. cholerae* (Garg *et al*., 2003; Kotetishvili *et al*., 2003; Thompson *et al*., 2008; Mohapatra *et al*., 2009; Teh *et al*., 2011; Thompson *et al*., 2011) and one virulence-associated gene, *toxR*. The seven-loci based MLST scheme published by Octavia et al in 2013 (Octavia *et al*., 2013) would have been a worthy alternative, but was not available when starting our study (2012).

#### DNA extraction

For DNA extraction, the isolates were boiled at 99°C for 10 min at 400 rpm in a Thermomixer comfort (Eppendorf, Hamburg, Deutschland) and centrifuged at 12 000 × *g* for 10 min at 4°C. The supernatant was diluted 1:10 with water (DEPC treated, sterile filtered; Sigma-Aldrich, St. Louis, MO, USA) and used as DNA template for PCR. The degenerate primer-sequences, amplicon sizes, annealing temperatures and references for the different PCRs are listed in Table 4 (length and position based on *V. cholerae* O1 biovar ElTor str. N16961, available at GenBank Accession Number NC_002505).

#### MLSA gene amplification

Each PCR mastermix contained 2.5 μL reaction buffer (0.8 M Tris-HCl, 0.2 M (NH_4_)_2_SO_4_; Solis Biodyne), 2 μL MgCl_2_ (final concentration 2 mM; Promega), forward and reverse primer (final concentrations: *recA*: 2 μM, *gyrB*: 0.48 μM, *pyrH*: 3 μM, *toxR*: 0.48 μM and *rpoA*: 0.48μM; Thermo Fisher), 0.5 μL desoxynucleoside triphosphates (0.4 mM; Solis BioDyne), 0.4 μL HOT FIREP® DNA polymerase (5 U μL^−1^) (Solis BioDyne), 5.0 μL template DNA and water (DEPC treated) to a final volume of 25 μL per reaction. The thermal program was set as 15 min at 95°C, then 30 cycles of 1 min at 95°C, 1 min at primer-specific annealing temperature (50°C for *recA* and *gyrB*, 55°C for *pyrH* and *toxR* and 53°C for *rpoA*) and 2 min at 72°C and finally 10 min at 72°C. PCR products were separated by 2% agarose gel electrophoresis.

#### Sequencing

The PCR products were purified and sequenced at Microsynth (Balgach, Switzerland). Both direction sequencing, forward and reverse, was performed for about 150 isolates. Raw sequence data (.ab1 files) were imported into CLC Main Workbench (Version 6.8.1-3, CLC bio). Consensus sequences were determined using forward and reverse sequences. Positions with conflicting data were located, the chromatogram reviewed and manually corrected. The chromatograms of sequences from one-directional sequencing were reviewed. At ambiguous positions, caused for example by a large pulse of dye overwriting the underlying base (occurring about once per ten sequences), the trace data were visually inspected and the base corrected. As the resulting forward and reverse sequences of these samples matched perfectly in nearly all cases, the remaining PCR products were sequenced in one direction only.

The overlapping region of all consensus sequences was determined by aligning and subsequently all sequences of one locus were trimmed manually to equal length. Table S1 summarizes the final lengths of the phylogenetic markers used for this MLSA approach, compared with the total length of the corresponding gene and the size of the PCR product. The four loci of each isolate were then concatenated. The order was set according to the distribution on chromosome 1 of *V. cholerae* O1 biovar ElTor str. N16961. For each reference strain, the four chosen genes were obtained from NCBI Gen-Bank database, trimmed to the previously determined individual start and end positions and concatenated. All concatenated DNA sequences were aligned by using the muscle algorithm (Edgar, 2004) in MEGA, Version 5.2 (Tamura *et al*., 2011) with the default alignment parameters, and the final alignment was exported as NEXUS file. The sequences determined in this study were deposited in GenBank under accession numbers KP833629–KP834308.

### Biodiversity analysis

Individual *V. cholerae* sequence types were computed with the software DnaSP 5.10.01 (Rozas, 2009). Venn diagrams were created by the tool VENNY (Oliveros, 2007) that is an interactive tool for comparing lists of values. To estimate the sequence type richness of *V. cholerae* at the three habitats in the lake, rarefaction curves were generated after analysis of roughly 100 isolates per each habitat (299 isolates in total) as well as for the enlarged data set containing additional 173 strains from the reed stand habitat. For this purpose, Chao1 matrices for each of the three sampling sites were composed individually, based on the concatenated maximum likelihood tree, and imported into EstimateS (Colwell, 2013).

The alpha-diversity indexes that characterize species richness and evenness were calculated as follows. Log Shannon biodiversity index was used to evaluate the within-community species diversity, which appears as the product of evenness and the number of species (Shannon and Weiner, 1963). It measures the likelihood that the next individual will be the same species as the previous sample. Given a sample size with many (more than 5) species, a value near 0 would indicate that every species in the sample is the same, whereas a value near or 4.6 would indicate that the number of individuals is evenly distributed between the five species. Berger–Parker Dominance index (May, 1975) that measures the numerical importance of the most abundant species was calculated as *d* = *N*_max_/*N* where *N*_max_ is the number of individuals in the most abundant species, and *N* is the total number of individuals in the sample.

### Phylogenetic analysis

The alignment file (FASTA format) was the source for the subsequent phylogenetic analysis. The alignment did not contain any ambiguous areas. Basically, the phylogenetic analysis was done as described in Druzhinina *et al*. (2010). The possibility of intragenic recombination, which would prohibit the use of the respective loci for phylogenetic analysis, was tested by LD-based statistics as implemented in DnaSP 4.50.3 (Rozas, 2009). The neutral evolution of the used fragments (*gyrB*, *recA*, *pyrH* and *toxR*) was tested by Tajima’s *D* test implemented in the same software. The interleaved NEXUS files were formatted from FASTA file using PAUP*4.0b10 (Swofford, 2002). The unconstrained GTR + I + G substitution model was applied to all sequence fragments. Two alternative phylogenetic trees were produced and only those relationships were considered significant that were supported by both approaches. Maximum likelihood phylogenetic trees were constructed with RAxML 8.0 using the GTR (general time reversible) model with gamma rate heterogeneity (Kirchberger *et al*., 2014; Stamatakis, 2014). Statistical support of branches from 1000 rapid bootstraps was mapped on the best scoring tree (bootstrap value >50), a bootstrap value of >70 was considered significant. For the construction of the Bayesian tree, Metropolis-coupled Markov chain Monte Carlo (MCMC) sampling was performed using MrBayes v. 3.0B4 (Ronquist and Huelsenbeck, 2003) with two simultaneous runs of four incrementally heated chains that performed 1 or 10 million generations, depending on the input data. The length of run (number of generations) for each dataset was determined using AWTY graphical system (Nylander *et al*., 2008) to check the convergence of MCMC. Bayesian posterior probabilities (PP) were obtained from the 50% majority rule consensus of trees sampled every 100 generations after removing the first trees using the ‘burnin’ command. Number of discarded generations was determined for every run based on visual analysis of the plot showing generation versus the log probability of observing the data. According to the protocol of Leache and Reeder (2002), *P* values higher than 0.94 were considered significant. The phylogenetic trees were visualized in TreeView (Page, 1996) and then labeled using vector graphic software.

### Analysis of recombination

Recombination within isolates from each individual habitat was tested by multiple tools as described in Druzhinina *et al*. (2008). First, the Partition Homogeneity Test (PHT) integrated in PAUP*4.0b10 (Swofford, 2002) was used to estimate the congruence among different loci datasets (Cunningham, 1997). For this test, heuristic search under parsimony optimality criterion was applied, parsimony-uninformative characters were excluded, gaps were treated as missing, and 10 000 repetitions were performed. A maximum of 100 trees was saved to conserve memory. Second, recombination analysis based on the maximum Chi-square test of Smith (Smith, 1992), LD *r*^2^ (Hill and Robertson, 1968), and LDD0 (LD versus distance |*D*|; Lewontin, 1964) as implemented in RecombiTEST package available on http://www.lifesci.sussex.ac.uk/CSE/test were performed. Third, the criterion of incongruence among the four gene genealogies was used to infer the occurrence of recombination among isolates, using the phi-test implemented in SplitsTree (Huson, 1998), which uses the pairwise homoplasy index, phi (=*Φ*) statistic, to detect refined incompatibility indicating recombination (Bruen *et al*., 2006). Fourth, we also performed a visual analysis of the topologies of phylogenetic trees.

### Statistical data analysis

One-way or main-effect ANOVAs were used to compare the three habitats. Tukey’s honest significant difference (Tukey HSD) test as implemented in STATISTICA 6.1 (StatSoft, Tulsa, OK) was used for post hoc comparisons to detect the contribution of each variable to the main effect of the F test resulting from the ANOVA. The summed data matrixes also were evaluated following factor analysis and multidimensional scaling to detect additional relationships between variables. Cluster analysis (Hartigan, 1975) was used to detect groups in the data set.

## Supplementary Material

Suppl. Info

## Figures and Tables

**Table 1:** Environmental variables and *V. cholerae* concentrations at the three habitats of the lake Neusiedler See.

Habitat	*n*		*V. cholerae* water (CFU L^−1^)	*V. cholerae* zooplankton (CFU L^−1^)	Total cell number (cells mL^−1^)	Copepods (Ind m^−3^)	Cladocerans (Ind m^−3^)	TSS^a^ (mg L^−1^)	DOC^b^ (mg L^−1^)	250/365 nm absorbance ratio	Ptot^c^ (μg L^−1^)	Chl. a^d^ (μg L^−1^)	pH value	Electr. Conductivity (mS cm^−1^)	Temp^e^ (°C)
Reed stand	33	Average	15 524	155	1.13 × 10^7^	8916	6384	20.4	22.3	8.3	36	7.9	8.5	2.2	17.2
		Median	6800	0	1.03 × 10^7^	3058	1626	16.5	22.3	8.1	34	7.2	8.5	2.2	18.1
		Min	0	0	3.13 × 10^6^	72	214	11.7	10.3	5.8	23	3.1	8.3	1.6	0.7
		Max	84 000	1419	2.95 × 10^7^	49 590	32 361	88.7	35.8	11.0	64	17.5	8.9	3.1	28.3
Open water	17	Average	12 208	266	8.89 × 10^6^	6029	8843	109.9	14.1	16.2	87	15.9	8.8	2.1	17.0
		Median	457	0	8.32 × 10^6^	5656	5767	62.3	14.0	15.8	75	15.3	8.8	2.0	18.8
		Min	0	0	4.54 × 10^6^	518	9	27.3	12.7	13.3	22	4.7	8.7	1.4	1.6
		Max	54 000	3194	1.58 × 10^7^	12 129	43 253	326.9	15.5	20.4	196	30.5	8.9	3.0	27.5
Intermediate habitat	13	Average	22 446	4497	9.13 × 10^6^	9617	10 172	81.9	17.4	15.4	58	10.0	8.8	2.2	18.4
		Median	6100	24	9.07 × 10^6^	10398	7885	56.0	17.6	15.0	56	7.0	8.9	2.2	19.6
		Min	0	0	3.08 × 10^6^	1815	12	30.0	15.4	11.0	16	3.6	8.7	1.8	7.6
		Max	90 000	46 272	1.23 × 10^7^	23 604	26 069	202.2	19.2	21.7	132	20.8	8.9	3.1	27.0

The 250/365 nm absorbance ratio is an indicator for total aromaticity and the presence of humic substances. A lower value indicates a higher concentration of high molecular weight substances (Kirschner *et al*., 2008).

a Total suspended solids.

b Dissolved organic carbon.

c Total phosphorus.

d Chlorophyll a.

e Temperature.

**Table 2:** Nucleotide properties of used loci and details of phylogenetic analysis.

	Phylogenetic marker				
	
Parameters	*gyrB*	*recA*	*toxR*	*pyrH*	Concatenated dataset
Fragment characterization	DNA gyrase subunit B	Recombinase A	Cholera toxin transcriptional activator	Uridylate kinase	Not applicable
Number of sequences	555	555	555	555	555
Number of characters: total/pars. inform./constant	487/57/410	648/167/477	658/140/497	477/38/432	2270/413/1816
Parameters of MCMC analysis					
Mean nt frequencies* A/C/G/T	0.27/0.22/0.26/0.5	0.26/0.22/0.27/0.25	0.31/0.24/0.21/0.24	0.21/0.20/0.30/0.29	Not applicable
Substitution rates* A–C/A–G/A–T/C–G/C–T/G–T	0.05/0.29/0.08/0.08/0.40/0.10	0.05/0.39/0.02/0.14/0.30/0.10	0.10/0.37/0.06/0.07/0.29/0.11	0.02/0.06/0.05/0.02/0.82/0.03	Not applicable
Number of generations/discarded first generations	1 000 000/700	1 000 000/1000	1 000 000/500	1 000 000/500	10 000 000/700
Total tree length	63.23	62.25	62.01	60.45	62.23
DNA polymorphism analysis and neutrality analysis					
Number of haplotypes	170	170	170	170	170
Number of sites excluding gaps and missing data	487	648	653	477	2265
Segregation sites/number of mutations	76/87	171/214	157/177	42/49	290/59
Nucleotide diversity, Pi	0.0242	0.02324	0.02421	0.00906	0.851/0.01886
Tajima’s *D* (NonSyn/Syn/ratio)	–1.47/–0.61/2.41	–2.27/–1.83/1.24	–1.95/–1.24/1.54	–1.076/–1.39/0.772	Not applicable
Tajima’s *D* test	n.s. (–0.70623, *P* > 0.1)	–1.905, *P* < 0.05	n.s. (–1.56513, *P* > 0.05)	n.s. (–1.49561, *P* > 0.1)	

**Table 3:** Diversity indexes of *V. cholerae* for the three habitats of the lake Neusiedler See.

	Open water	Intermediate habitat	Reed stand
Total number of organisms	99	100	273 (100)
Sequence types	19	18	84 (32)
Average population size	5.21	5.56	3.26 (3.13)
Shannon index, log	2.56	2.82	5.48 (4.68)
Berger–Parker Dominance index	0.586	0.510	0.146 (0.1)

For the reed stand, the values in brackets indicate the results of the small data set (100 strains isolated on a single day in July 2012).

**Table 4:** Primers used for this multilocus sequence analysis.

Primer	Sequence (5′–3′)	Position	Fragment length (nt)	Reference	PCR annealing temp. (°C)
gyrB F	GAAGGBGGTATTCAAGC	655	629	(Garg *et al*., 2003)	50
gyrB R	GAGTCACCCTCCACWATGTA	1283		(Kotetishvili *et al*., 2003)	
recA-01-F	TGARAARCARTTYGGTAAAGG	222	837	(Thompson *et al*., 2011)	50
recA-02-R	TCRCCNTTRTAGCTRTACC	1058			
toxR-F	CCTTCGATCCCCTAAGCAATAC	80	779	(Rivera *et al*., 2001)	55
toxR-R	AGGGTTAGCAACGATGCGTAAG	858			
pyrH-04-F	ATGASNACBAAYCCWAAACC	1	617	(Thompson *et al*., 2005; Thompson *et al*., 2008; Thompson *et al*., 2011)	55
pyrH-02-R	GTRAABGCNGMYARRTCCA	617			
rpoA-01-F	ATGCAGGGTTCTGTDACAG	1	970	(Thompson *et al*., 2005; Thompson *et al*., 2008; Thompson *et al*., 2011)	53
rpoA-03-R	GHGGCCARTTTTCHARRCGC	970			
ompW-F	CACCAAGAAGGTGACTTTATTGTG	64	304	(Nandi *et al*., 2000)	59
ompW-R	GGTTTGTCGAATTAGCTTCACC	367			
