# Supplementary material for: High genetic diversity of Vibrio cholerae in the European lake Neusiedler See is associated with intensive recombination in the reed habitat and the long-distance transfer of strains
Source: Environ Microbiol. Author manuscript; Available in PMC 2017 Dec 6. (PMC5718291; doi:10.1111/1462-2920.13612)
Supplement: Suppl. Info [file NIHMS75203-supplement-Suppl__Info.pdf]

Pretzer C, Druzhinina IS, Amaro C, Benediktsdóttir E, Hedenström I, Hervio-Heath D, Huhulescu S, Schets FM, Farnleitner AH, Kirschner AKT. High genetic diversity of *Vibrio cholerae* in the European lake Neusiedler See is associated with intensive recombination in the reed habitat and the long-distance transfer of strains. *Environmental Microbiology*

SUPPLEMENTAL INFORMATION

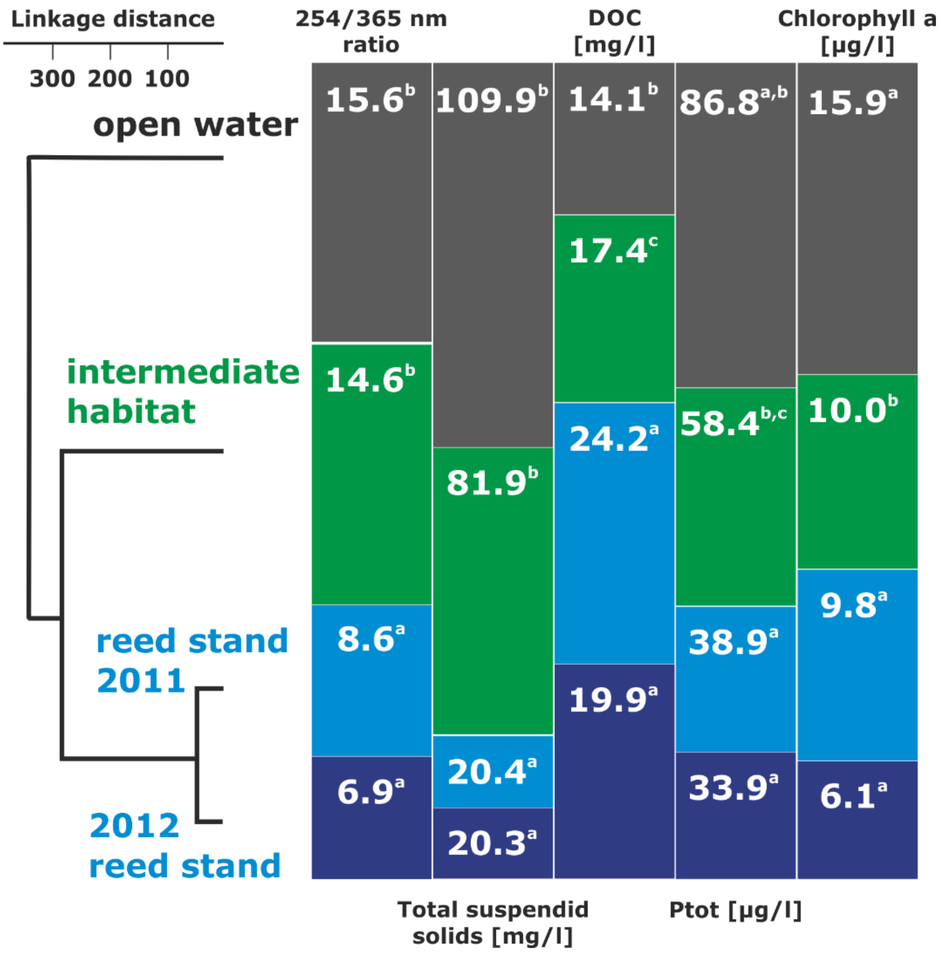

Figure S1:

Cluster analysis of the three investigated habitats based on their ecological characteristics. a–c-values with the same letter are not significantly different (ANOVA, post hoc Tukey HSD test, $P < 0.05$).

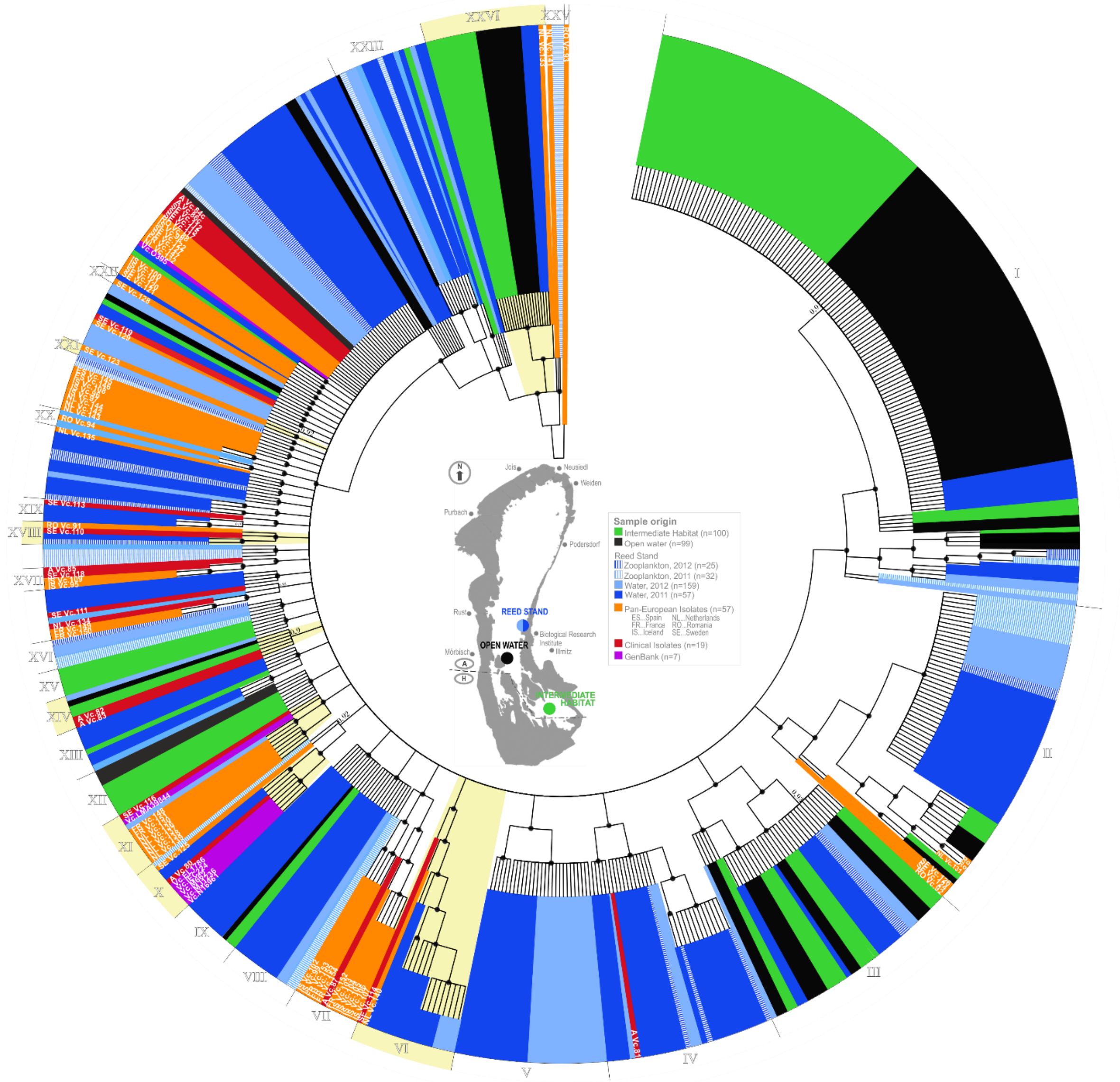

Figure S2: Bayesian tree resulting from analysis of the concatenated sequences comprising of *gyrB*, *recA*, *pyrH* and *toxR* [2,270 bp] of 555 *Vibrio cholerae* isolates including 472 of lake Neusiedler See, 57 Pan-European isolates, 7 reference strains and 19 clinical isolates. Nodes indicated by black circles correspond to HTU supported by posterior probabilities of > 0.94 as inferred based on the Bayesian analysis (Figure S2). Numerical values at nodes indicate bootstrap support obtained after 1000 replications. Supported clades were numbered with Roman numerals but do not correspond to clades of maximum likelihood (ML) analysis in the main manuscript (**Figure 2**). Clades marked with yellow indicated clades with mixed local and remote isolates and with mixed local environmental and clinical isolates that were significantly supported by ML. Colors denote origin of isolate detailed on the insert. Insert depicts a schematic map of the lake, location of studied habitats and the isolation substrate for the isolates. Branch lengths are transformed to equal length for better visualization and do not represent true phylogenetic distances.

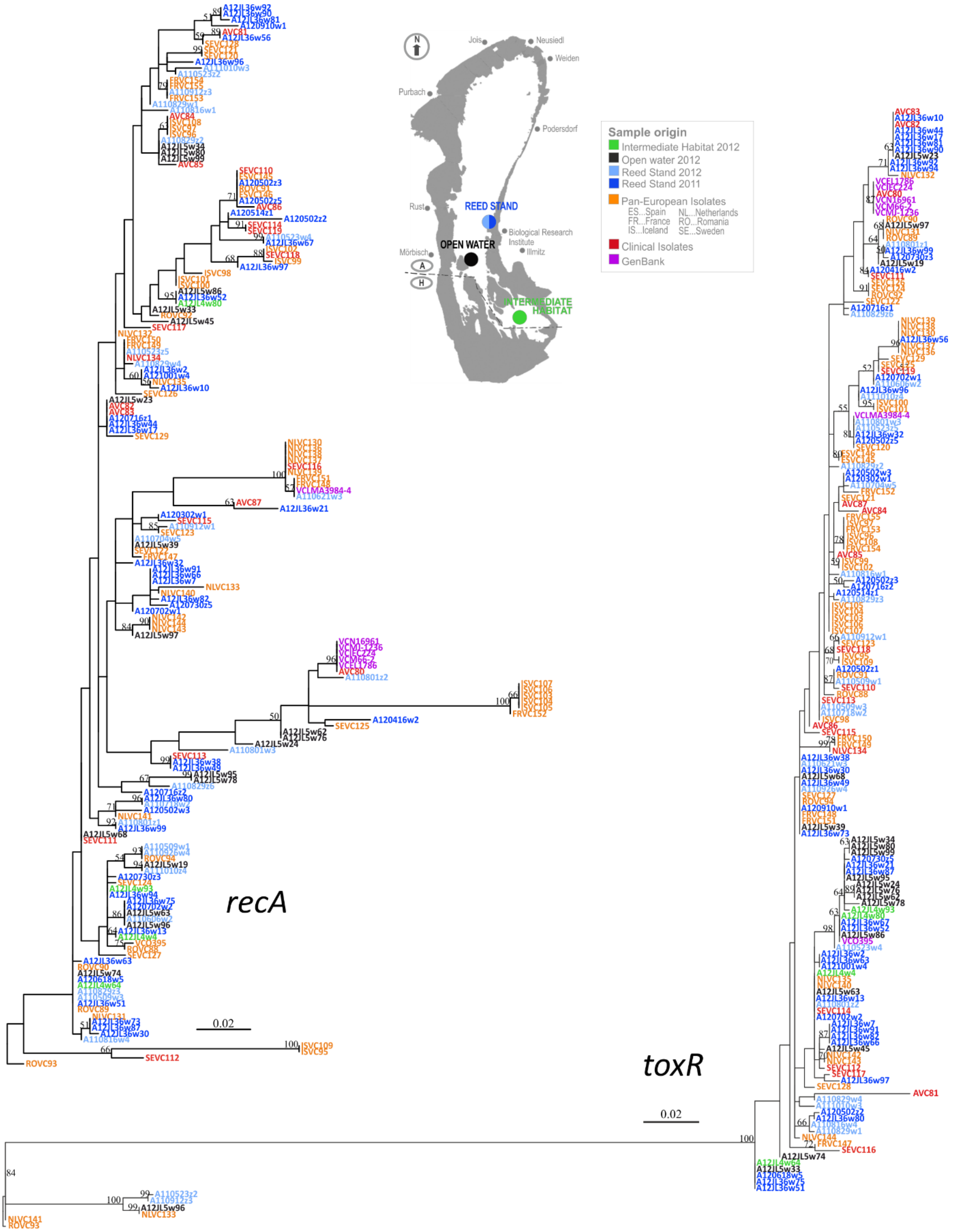

Figure S3: Single locus trees for *recA* and *toxR*. Phylogenetic tree based on the maximum likelihood method resulting from analysis of the *recA and toxR* gene fragments of 177 *Vibrio cholerae* isolates including 94 of lake Neusiedler See, 57 Pan-European isolates, 7 reference strains and 19 clinical isolates. Bootstrap values after 1000 replications are shown. Colors denote origin of isolate detailed on the insert. Insert depicts a schematic map of the lake and location of studied habitats.

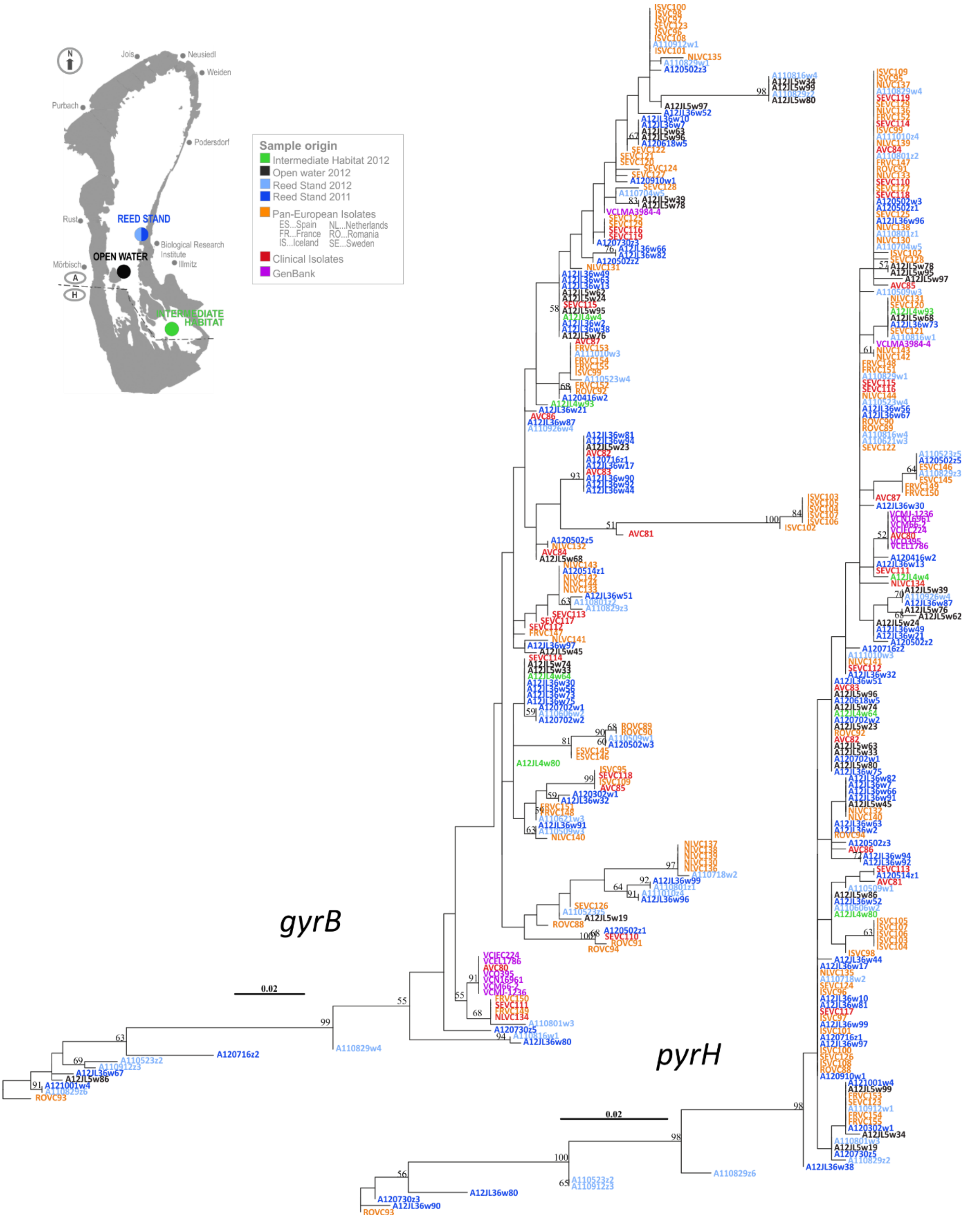

Figure S4: Single locus trees for *gyrB and pyrH*. Phylogenetic tree based on the maximum likelihood method resulting from analysis of the *gyrB and pyrH* gene fragments of 177 *Vibrio cholerae* isolates including 94 of lake Neusiedler See, 57 Pan-European isolates, 7 reference strains and 19 clinical isolates. Bootstrap values after 1000 replications are shown. Colors denote origin of isolate detailed on the insert. Insert depicts a schematic map of the lake and location of studied habitats.

Table S1: Gene length, PCR product size and the fragment used for the analysis.

Gene	Gene size [nt]	PCR product [nt]	Fragment for analysis [nt]
gyrB	2,418	629	487
recA	1,065	837	648
toxR	885	779	658
pyrH	732	617	477
rpoA	993	970	-
		Concatenated sequence	2,270

Table S2: Summarized sample set. Sample source, collection year in brackets, and number of isolates.

Austria, lake Neusiedler See						
Intermediate habitat	Open water	Reed stand		Austria clinical	International samples	References GenBank
		water	zooplankton			
(2012)	(2012)	(2011-2012)		(2008-2012)	(1997-2013)	(1937-2010)
100	99	216	57	8	68	7

Table S3: Isolates used in this study.

Quantity	Isolates numbers	Date of Isolation	Place of Isolation	Source
			**AUSTRIA ENVIRONMENTAL**	
299	**A12JL4w1 to A12JLw100**	2012-09-07	Austria, lake Neusiedler See, Intermediate habitat	Water
	**A12JL5w1to A12JL5w99**		Austria, lake Neusiedler See, Open water	
	**A12JL36w1 to A12JL36w100**		Austria, lake Neusiedler See, Reed stand	
57	**A110509w1 to A110509w5**	2011-05-09	Austria, lake Neusiedler See, Reed stand	Water
	**A110523w1 to A110523w5**	2011-05-23		
	**A110606w1 to A110606w2**	2011-06-06		
	**A110621w1 to A110621w5**	2011-06-21		
	**A110704w1 to A110704w5**	2011-07-04		
	**A110718w1 to A110718w5**	2011-07-18		
	**A110801w1 to A110801w5**	2011-08-01		
	**A110816w1 to A110816 w5**	2011-08-16		
	**A110829w1 to A110829 w5**	2011-08-29		
	**A110912w1 to A110912w5**	2011-09-12		
	**A110926w1 to A110926 w5**	2011-09-26		
	**A111010w1 to A111010w5**	2011-10-10		
59	**A120302w1**	2012-03-02		
	**A120416w1 to A120416w3**	2012-04-16		
	**A120502w1 to A120502w5**	2012-05-02		
	**A120514w1 to A120514w5**	2012-02-14		
	**A120605w1 to A120605w5**	2012-06-05		
	**A120618w1 to A120618w5**	2012-06-18		
	**A120702w1 to A120702w5**	2012-07-02		
	**A120716w1 to A120716 w5**	2012-07-16		
	**A120730w1 to A120730w5**	2012-07-30		
	**A120806w1 to A120806w5**	2012-08-06		
	**A120828w1 to A120828w5**	2012-08-28		
	**A120910w1 to A120910w5**	2012-09-10		
	**A121001w1 to A121001w5**	2012-10-01		
32	**A110523z1 to A110523z5**	2011-05-23	Austria, lake Neusiedler See, Reed stand	Zooplankton
	**A110606z1 to A110606z4**	2011-06-06		
	**A110621z1 to A110621z3**	2011-06-21		
	**A110801z1 to A110801 z5**	2011-08-01		
	**A110829z1 to A110829z6**	2011-08-29		
	**A110912z1 to A110912 z3**	2011-09-12		
	**A110926z1 to A110926z2**	2011-09-26		
	**A111010z1 to A111010z4**	2011-10-10		
25	**A120502z1 to A120502z5**	2012-05-02		
	**A120514z1 to A120514z5**	2012-05-14		
	**A120618z1 to A120618z5**	2012-06-18		
	**A120716z1 to A120716 z5**	2012-07-16		
	**A120730z1 to A1207030z5**	2012-07-30		
			**AUSTRIA, CLINICAL**	
8	**A *(Vc.* 80)**	2008	Austria	Human (O1 Ogawa (1))
	**A *(Vc.* 81)**	2008	Austria	Human
	**A *(Vc.* 82)**	2008	Austria	Human
	**A *(Vc.* 83)**	2009	Austria	Human
	**A *(Vc.* 84)**	2009	Austria	Human
	**A *(Vc.* 85)**	2010	Austria	Human
	**A *(Vc.* 86)**	2011	Austria	Human
	**A *(Vc.* 87)**	2012	Austria	Human (O1 Inaba, Egypt)
			**ROMANIA, ENVIRONMENTAL**	
7	**RO *(Vc.* 88)**	1997	Romania, Braila	Human faeces
	**RO *(Vc.* 89)**	2000	Romania, Galati	Danube water
	**RO *(Vc.* 90)**	2000	Romania, lake Brates (near Galati)	Lake water
	**RO *(Vc.* 91)**	2001	Romania, Galati	Human faeces
	**RO *(Vc.* 92)**	2001	Romania, lake Siutghiol (near Mamaia)	Lake water
	**RO *(Vc.* 93)**	2002	Romania, Galati	Wastewater
	**RO *(Vc.* 94)**	2002	Romania, lake Costinesti	Lake water
			**ICELAND, ENVIRONMENTAL**	
15	**IS *(Vc.* 95)**	2011-08-27	Iceland, Berserkseyri, West coast of Iceland, rural area	Sediment
	**IS *(Vc.* 96)**	2011-01-19	Iceland, Coast beneath the street ÆgissÍða, ReykjavÍk	Sea water
	**IS *(Vc.* 97)**	2011-01-19	Iceland, Coast beneath the street ÆgissÍða, ReykjavÍk	Kelp
	**IS *(Vc.* 98)**	2011-08-27	Iceland, Berserkseyri, West coast of Iceland, rural area	Sea water
	**IS *(Vc.* 99)**	2011-08-27	Iceland, Berserkseyri, West coast of Iceland, rural area	Kelp
	**IS *(Vc.* 100)**	2011-01-19	Iceland, Coast beneath the street ÆgissÍða, ReykjavÍk	Burrowing lugworm
	**IS *(Vc.* 101)**	2011-03-21	Iceland, Coast beneath the street ÆgissÍða, ReykjavÍk	Sediment

	**IS *(Vc.* 102)**	2011-08-27	Iceland, Skarðshver, Northern Iceland, rural area	Amphipods
	**IS *(Vc.* 103)**	2012-05-22	Iceland, Skarðshver, Northern Iceland, rural area	Sea water
	**IS *(Vc.* 104)**	2012-05-22	Iceland, Skarðshver, Northern Iceland, rural area	Kelp
	**IS *(Vc.* 105)**	2012-05-22	Iceland, Skarðshver, Northern Iceland, rural area	Kelp
	**IS *(Vc.* 106)**	2012-05-22	Iceland, Skarðshver, Northern Iceland, rural area	Sea water
	**IS *(Vc.* 107)**	2011-11-22	Iceland, Skarðshver, Northern Iceland, rural area	Sediment
	**IS *(Vc.* 108)**	2012-06-14	Iceland, Coast beneath the street ÆgissÍða, ReykjavÍk	Sea water
	**IS *(Vc.* 109)**	2012-06-21	Iceland, Berserkseyri, West coast of Iceland, rural area	Lugworm
			**SWEDEN, CLINICAL**	
	**SE *(Vc.* 110)**	2006-07	Sweden	Human, blood
	**SE *(Vc.* 111)**	2006-07	Sweden	Human, wound
	**SE *(Vc.* 112)**	2006-07	Sweden	Human, blood
	**SE *(Vc.* 113)**	2006-07	Sweden	Human, wound
10	**SE *(Vc.* 114)**	2006-08	Sweden	Human, ear
	**SE *(Vc.* 115)**	2006-08	Sweden	Human, nasopharynx
	**SE *(Vc.* 116)**	2006-08	Sweden	Human, ear
	**SE *(Vc.* 117)**	2006-08	Sweden	Human, ear
	**SE *(Vc.* 118)**	2006-08	Sweden	Human, blood
	**SE *(Vc.* 119)**	2006-08	Sweden	Human, ear
			**SWEDEN, ENVIRONMENTAL**	
	**SE *(Vc.* 120)**	2006-07	Sweden, pond for irrigation (connected to SE *Vc.*111)	
	**SE *(Vc.* 121)**	2006-07	Sweden, Southern Baltic sea	
	**SE *(Vc.* 122)**	2006-07	Sweden, Southern Baltic sea	
	**SE *(Vc.* 123)**	2006-07	Sweden, Southern Baltic sea	
10	**SE *(Vc.* 124)**	2006-07	Sweden, Southern Baltic sea	Water
	**SE *(Vc.* 125)**	2006-07	Sweden, Southern Baltic sea	
	**SE *(Vc.* 126)**	2006-07	Sweden, fresh water lake	
	**SE *(Vc.* 127)**	2006-08	Sweden, Southern Baltic sea	
	**SE *(Vc.* 128)**	2006-08	Sweden, Southern Baltic sea	
	**SE *(Vc.* 129)**	2006-08	Sweden, fresh water lake	
			**NETHERLANDS, ENVIRONMENTAL**	
	**NL *(Vc.* 130)**	2009	Netherlands, Binnenschelde	Water
	**NL *(Vc.* 131)**	2009	Netherlands, Binnenschelde	Water
	**NL *(Vc.* 132)**	2009	Netherlands, Binnenschelde	Water
	**NL *(Vc.* 133)**	2009	Netherlands, Binnenschelde	Water
	**NL *(Vc.* 134)**	2009	Netherlands	Human
	**NL *(Vc.* 135)**	2010	Netherlands, Binnenschelde	Water
	**NL *(Vc.* 136)**	2010	Netherlands, Binnenschelde	Water
15	**NL *(Vc.* 137)**	2010	Netherlands, Binnenschelde	Water
	**NL *(Vc.* 138)**	2010	Netherlands, Binnenschelde	Water
	**NL *(Vc.* 139)**	2011	Netherlands, Binnenschelde	Water
	**NL *(Vc.* 140)**	2011	Netherlands, Binnenschelde	Water
	**NL *(Vc.* 141)**	2011	Netherlands, Wadden Sea, North Sea	Water
	**NL *(Vc.* 142)**	2012	Netherlands, Wadden Sea, North Sea	Water
	**NL *(Vc.* 143)**	2012	Netherlands, Wadden Sea, North Sea	Water
	**NL *(Vc.* 144)**	2012	Netherlands, Binnenschelde	Water
			**SPAIN, ENVIRONMENTAL**	
2	**ES *(Vc.* 145)**	2013-06-19	Spain, Natural Park of Delta del Ebro, Alfacada pond	skin mucus of wild-eels
	**ES *(Vc.* 146)**	2013-06-19	Spain, Natural Park of Delta del Ebro, Alfacada pond	
			**FRANCE, ENVIRONMENTAL**	
	**FR*)(Vc)*147)**	1999	France, Charente Maritime, 17, Atlantic coast	Mussel, *Mytilus edulis*
	**FR *(Vc.* 148)**	2012		
	**FR *(Vc.* 149)**	2012		
	**FR *(Vc.* 150)**	2012		
9	**FR *(Vc.* 151)**	2012	France, Finistère, 29, Atlantic coast	Clams, *Ruditapes decussatus*
	**FR *(Vc.* 152)**	2013		
	**FR *(Vc.* 153)**	2013		
	**FR *(Vc.* 154)**	2013		
	**FR *(Vc.* 155)**	2013		
			**REFERENCE STRAINS***	
	***Vibrio cholerae* M66-2**	1937	Indonesia	Human
	***Vibrio cholerae* O395**	1965	India	Human
	***Vibrio cholerae* O1 biovar ETor str. N16961**	1971	Bangladesh	Human
7	***Vibrio cholerae* MJ-1236**	1994	Bangladesh	Human
	***Vibrio cholerae* IEC224**	1994	Brazil (2)	Human
	Vibrio cholerae LMA3984-4	2007	Brazil, Tucunduba Igarapé, Amazonian tributary (3)	Water
	Vibrio cholerae O1 str. 2010EL-1786	2010	Artibonite, Haiti (4)	Human
**Total 555**				

Literature to Table S3

(1) Huhulescu S, Leitner E, Feierl G, Allerberger F. 2010. Laboratory-acquired Vibrio cholerae O1 infection in Austria, 2008. Clin Microbiol Infect 16:1303-1304

(2) de Sa Morais LL, Garza DR, Loureiro EC, Nunes KN, Vellasco RS, da Silva CP, Nunes MR, Thompson CC, Vicente AC, Santos EC. 2012. Complete genome sequence of a sucrose-nonfermenting epidemic strain of Vibrio cholerae O1 from Brazil. J Bacteriol 194:2772.

(3) Perez Chaparro PJ, McCulloch JA, Cerdeira LT, Al-Dilaimi A, Canto de Sa LL, de Oliveira R, Tauch A, de Carvalho Azevedo VA, Cruz Schneider MP, da Silva AL. 2011. Whole genome sequencing of environmental Vibrio cholerae O1 from 10 nanograms of DNA using short reads. J Microbiol Methods 87:208-212.

(4) Reimer AR, Van Domselaar G, Stroika S, Walker M, Kent H, Tarr C, Talkington D, Rowe L, Olsen-Rasmussen M, Frace M, Sammons S, Dahourou GA, Boncy J, Smith AM, Mabon P, Petkau A, Graham M, Gilmour MW, Gerner-Smidt P; V. cholerae Outbreak Genomics Task Force. 2011. Comparative genomics of Vibrio cholerae from Haiti, Asia, and Africa. Emerg Infect Dis 17:2113-2121.
